# Small molecule kinase inhibitors enhance aminolevulinic acid-mediated protoporphyrin IX fluorescence and PDT response in triple negative breast cancer cell lines

**DOI:** 10.1117/1.JBO.26.9.098002

**Published:** 2021-09-20

**Authors:** Pratheeba Palasuberniam, Daniel Kraus, Matthew Mansi, Richard Howley, Alexander Braun, Kenneth A. Myers, Bin Chen

**Affiliations:** a University of the Sciences, Philadelphia College of Pharmacy, Department of Pharmaceutical Sciences, Philadelphia, Pennsylvania, United States; b University of the Sciences, Misher College of Arts and Sciences, Philadelphia, Pennsylvania, United States; c University of Pennsylvania, Perelman School of Medicine, Department of Radiation Oncology, Philadelphia, Pennsylvania, United States

**Keywords:** aminolevulinic acid, protoporphyrin IX, photodynamic therapy, ABCG2, lapatinib, ferrochelatase

## Abstract

**Significance:** We demonstrate that clinically used kinase inhibitors such as lapatinib can be used for enhancing aminolevulinic acid (ALA) for tumor fluorescence imaging and photodynamic therapy (PDT).

**Aim:** ALA is used as a prodrug for protoporphyrin IX (PpIX) fluorescence-guided tumor resection and PDT. Our previous studies indicate that tumors with high ABCG2 activity exhibit low PpIX fluorescence, which hampers the application of ALA. We aim to determine whether clinically used ABCG2-interacting kinase inhibitors increase ALA-PpIX fluorescence and PDT.

**Approach:** PpIX fluorescence was determined by spectrofluorometry, flow cytometry, and confocal microscopy after ALA alone or in combination with kinase inhibitors in triple negative breast cancer (TNBC) cell lines. Cytotoxicity was examined after ALA-PDT alone or in combination with kinase inhibitors. Effect of single and combination treatments on apoptosis was assessed by Western blot.

**Results:** Four kinase inhibitors (lapatinib, PD169316, sunitinib, gefitinib) significantly increased ALA-PpIX fluorescence and PDT response in TNBC cells with ABCG2 activity, but not in MCF10A nontumor breast epithelial cell line without ABCG2 activity. Confocal microscopic imaging showed that PpIX fluorescence was weak and diffuse after ALA alone, which was greatly enhanced by kinase inhibitors, particularly in the mitochondria. Lapatinib was the only inhibitor that significantly reduced PpIX efflux in cell culture medium and showed stronger enhancement of PDT response than other kinase inhibitors. Lapatinib, in combination with ALA, induced tumor cells to undergo apoptosis, whereas no apoptosis was detected after each individual treatment.

**Conclusions:** Although all four kinase inhibitors were able to enhance ALA-PpIX fluorescence and PDT, lapatinib exhibited the strongest enhancement effect. As an FDA-approved kinase inhibitor for breast cancer treatment, lapatinib is ready to be used in combination with ALA for therapeutic enhancement in tumors with elevated ABCG2 activity. This rational combination approach warrants further investigation in tumor models.

## 1 Introduction

Aminolevulinic acid (ALA) is an FDA-approved drug for photodynamic therapy (PDT) and fluorescence-guided tumor resection.^1^ Although ALA itself has no fluorescence and photosensitizing activity, it is metabolized in the heme biosynthesis pathway to produce protoporphyrin IX (PpIX) in mitochondria that exhibits red fluorescence and photosensitizing activity.^2^ Catalyzed by ferrochelatase (FECH) in mitochondria, PpIX is further chelated with ferrous iron (Fe) to form the pathway final product heme with no fluorescence and photosensitizing activity. Although the mechanism is not yet clear, the preferential accumulation of PpIX in malignant cells enables the use of ALA as a prodrug for tumor treatment with PDT and tumor surgery under the guidance of PpIX fluorescence. PDT with ALA is now commonly used in clinic for precancer skin lesions and superficial skin cancers with good response rate and excellent cosmetic outcomes.^3^ ALA-PpIX fluorescence-guided surgery of high-grade gliomas results in better progression-free survival than conventional white light surgery by dissecting more tumor tissues.^4^ In addition to skin and brain tumors, ALA is currently under investigation for other types of tumors as a PDT agent and/or intraoperative imaging probe.

Because ATP-binding cassette subfamily G member 2 (ABCG2) is involved in the efflux transport of PpIX, inhibition of ABCG2 transporter activity represents a promising therapeutic strategy for the enhancement of ALA-PpIX fluorescence and PDT response.^2^ ABCG2 inhibitors such as fumitremorgin C (FTC) and its derivative Ko143 have been shown to enhance ALA-PpIX fluorescence and PDT effects in ABCG2-expressing cells.^5^^–^^7^ Tumor cell lines with elevated ABCG2 activity exhibited good responses to Ko143 for the enhancement of PpIX and PDT, but not tumor cell lines with little ABCG2 activity.^8^^,^^9^ These previous studies support the combination of ABCG2 inhibitors and ALA for therapeutic enhancement. However, FTC and Ko143 are not suitable for *in vivo* application due to neurotoxicity and the instability in serum, respectively.^10^^,^^11^ Despite extensive efforts, clinically useful ABCG2 inhibitors are still not available.^12^

Some clinically used kinase inhibitors for cancer treatment are ABCG2 substrates and, therefore, act as competitive inhibitors of the transporter.^13^ These ABCG2-interacting kinase inhibitors including imatinib^14^ and gefitinib^15^ were shown to increase ALA-PpIX fluorescence and PDT effects, suggesting that these drugs may be repurposed for the therapeutic enhancement of ALA. We have evaluated over a dozen small molecule kinase inhibitors and identified six FDA-approved drugs that significantly increased the intracellular PpIX in a renal cell carcinoma cell lines with high ABCG2 activity.^9^ Notably, lapatinib was the only one that not only increased intracellular PpIX but also reduced PpIX efflux in the medium. In this study, we further assessed some of these kinase inhibitors for the enhancement of ALA-PpIX fluorescence and PDT effect in triple negative breast cancer (TNBC) cell lines, which are known to be resistant to chemotherapeutics due to the elevation of ABCG2 and other efflux transporters.^16^ We showed that lapatinib and other kinase inhibitors greatly enhanced ALA-PpIX fluorescence in mitochondria and sensitized TNBC cells to undergo apoptosis induced by ALA-PDT, demonstrating the effectiveness of using these clinically available drugs for the enhancement of ALA.

## 2 Materials and Methods

### 2.1 Chemicals

5-ALA hydrochloride from Frontier Scientific Inc. (Logan, Utah) and rhodamine 123 from Life Technologies (Grand Island, New York) were dissolved in phosphate-buffered saline (PBS). Lapatinib, gefitinib, and sunitinib malate salt (all from LC Laboratories, Woburn, Massachusetts), PD169316 (from Millipore, Burlington, Massachusetts), pheophorbide a (Pha, from Frontier Scientific Inc.), Ko143 (from Santa Cruz Biotechnology, Santa Cruz, California) were all dissolved in DMSO. All chemicals were sterilized through 0.22-μm filters and stored in a −20°C freezer.

### 2.2 Cell Culture

MCF10A human breast epithelial cells were maintained in Dulbecco’s modified Eagle medium (DMEM)/Ham’s F-12 (50/50) medium supplemented with epidermal growth factor 20 ng/mL, insulin 10  μg/mL, hydrocortisone 0.5  μg/mL, cholera toxin 100 ng/mL, 5% horse serum (Atlanta Biologicals), and 1% antibiotics and antimycotics (Mediatech, Manassas, Virginia). Triple-negative breast tumor cell lines (HCC1395, BT-20, MDA-MB-231, and Hs578T) were cultured in RPMI 1640 (HCC1395), EMEM (BT-20), or DMEM (MDA-MB-231, Hs578T) medium supplemented with 9% fetal bovine serum (Atlanta Biologicals) and 1% antibiotics and antimycotics. All cell lines were purchased from American Type Culture Collection (ATCC, Manassas, Virginia). Cells were maintained at 37°C in a cell culture incubator with 5% ${\mathrm{CO}}_{2}$.

### 2.3 Spectrofluorometric Analysis of PpIX Fluorescence

Cells were implanted in six-well cell culture plates and allowed to grow for 2 days. Cells were incubated with ALA alone, Ko143 or kinase inhibitors alone, and ALA in combination with Ko143 or kinase inhibitors for 4 h in the complete medium. Cell culture medium was collected after the treatment. Cells were rinsed twice with PBS and lysed in 1% SDS solution. Both cell culture medium and lysates were centrifuged and the supernatants were collected for PpIX fluorescence measurement using a Fluoromax-3 fluorescence spectrometer (Horiba JY, Edison, New Jersey) with the excitation wavelength of 400±2.5  nm. Effects of inhibitors on PpIX fluorescence in cell lysates and medium were determined using the equation $(F_{\mathrm{ALA}+\text{inhibitor}}-F_{\text{Inhibitor}})/F_{\mathrm{ALA}}\times 100\%$ where $F_{\mathrm{ALA}+\text{inhibitor}},F_{\text{Inhibitor}}$, and $F_{\mathrm{ALA}}$ indicate the fluorescence of samples treated with ALA in combination with an inhibitor, inhibitor alone, and ALA alone, respectively.

### 2.4 Flow Cytometry

Cells were implanted in 60-mm dishes and allowed to grow for 2 days. Cells were incubated with ALA alone, Ko143 or kinase inhibitors alone, and ALA in combination with Ko143 or kinase inhibitors for 4 h in the complete medium. After the incubation, cells were rinsed with PBS and trypsinized. Cells were resuspended in PBS and measured with a FACSCalibur flow cytometer (BD Biosciences) for fluorescence in the FL3 channel (488 nm excitation, 650 nm long-pass emission). About 20,000 cells were measured and recorded for each experiment. Effects of inhibitors on cell fluorescence were determined using the equations described above.

### 2.5 ABCG2 Transporter Activity Assay

Cells were implanted in 60-mm dishes and allowed to grow for 2 days. Cells were incubated with ABCG2 substrate pheophorbide a (Pha, 500 nM) alone or in combination with ABCG2 inhibitor Ko143 (1  μM) for 4 h in the complete medium. Cells were rinsed with PBS after the incubation and trypsinized. Cells were resuspended in PBS and measured with a FACSCalibur flow cytometer for fluorescence in the FL3 channel. Fluorescence after Pha in combination with Ko143 was normalized to that after Pha treatment alone to indicate the ABCG2 activity.

### 2.6 FECH Activity Assay

Effects of kinase inhibitor lapatinib on FECH activity were determined as described previously.^9^ Briefly, cells in 100-mm dishes were treated with lapatinib (1  μM) for 4 h or untreated for control and lysed with 0.1% Triton X-100 in PBS containing 200  μM palmitic acid and 1× protease inhibitor cocktail. Lysates were centrifuged to obtain the supernatant, which was incubated with zinc acetate (500  μM) and various concentrations of PpIX for 10 min at 37°C. The resultant Zn-PpIX was detected using a Fluoromax-3 fluorescence spectrometer with the excitation wavelength of 400±2.5  nm and emission wavelength of 590 nm. FECH activity was indicated by the amount of Zn-PpIX produced per gram cell protein per minute.

### 2.7 Confocal Fluorescence Microscopy

Cells were implanted in glass bottom cell culture dishes (MatTek, Ashland, Massachusetts) and allowed to grow for 2 days. Cells were treated with ALA alone or in combination with Ko143 or kinase inhibitors for 4 h in the complete medium. Rhodamine 123 (Rho) was added to the medium at 30 min before imaging to highlight the mitochondria. After the incubation, cells were rinsed with PBS twice and incubated in serum-free DMEM medium for imaging. Live-cell imaging was performed with a Nikon TiE (Eclipse) confocal microscope using a 60×1.40  NA oil immersion objective as described previously.^17^ PpIX fluorescence was imaged with 405 nm laser excitation and 700±37.5  nm emission with the exposure time of 500 ms. Rho fluorescence was imaged with 488 nm laser excitation and 525±18  nm emission with the exposure time of 300 ms. Differential interference contrast images were acquired using exposure times in the range of 100 to 200 ms at the same magnification. The colocalization between PpIX and Rho fluorescence was determined by the Pearson’s coefficient analyzed with the NIH Image J software.

### 2.8 PDT Treatment and Cytotoxicity Assay

Cells were implanted in 96-well plates and allowed to grow for 2 days to reach around 70% confluency. Cells were incubated with complete medium containing no drug (for control), ALA alone, Ko143 or kinase inhibitors alone, and ALA in combination with Ko143 or kinase inhibitors for 4 h. After the incubation, cells were treated with $5\;{\mathrm{mW}/\mathrm{cm}}^{2}$ irradiance of 633 nm light for 10 or 20 min, resulting in a light fluence of 3 or $6\;{J/\mathrm{cm}}^{2}$. Light illumination was provided by a diode laser system (High Power Devices Inc., North Brunswick, New Jersey) coupled to a 600-μm core diameter optical fiber fitted with a microlens at the end of fiber to achieve homogeneous irradiation. Light intensity was measured with an optical power meter (Thorlabs, Inc., North Newton, New Jersey). Immediately after light treatment, drug-containing media were replaced with fresh media. Cell viability was determined at 24 h after treatment with CellTiter 96 Aqueous Non-Radioactive Cell Proliferation Assay (MTS assay, Promega, Madison, Wisconsin) by following the manufacturer’s instruction.

### 2.9 Western Blot

Cells were lysed at 70% to 80% confluency in NP40 lysis buffer supplemented with protease and phosphatase inhibitors as described previously.^18^ Cell lysates were separated by sodium dodecyl sulfate polyacrylamide gel electrophoresis and electrophoretically transferred to polyvinylidene fluoride membranes (Millipore). Blots were first incubated with the primary antibody for poly (ADP-ribose) polymerase (PARP) or actin and then with horseradish peroxidase-conjugated secondary antibody. All antibodies were obtained from Cell Signaling Technology (Danvers, Massachusetts). Immunoblots were incubated with SuperSignal West Dura extended duration substrate (Thermo Scientific) and immunoreactive bands were captured with GE Amersham Imager 600 (GE Healthcare Bio-Sciences, Piscataway, New Jersey).

### 2.10 Statistical Analysis

One-way or two-way ANOVA with posttests were used to determine statistical differences between groups and statistical significance was accepted at P<0.05. Statistical analysis was performed using GraphPad Prism software (La Jolla, California).

## 3 Results

### 3.1 Kinase Inhibitors Increased ALA-PpIX in TNBC Cell Lines

**Fig. 1:**
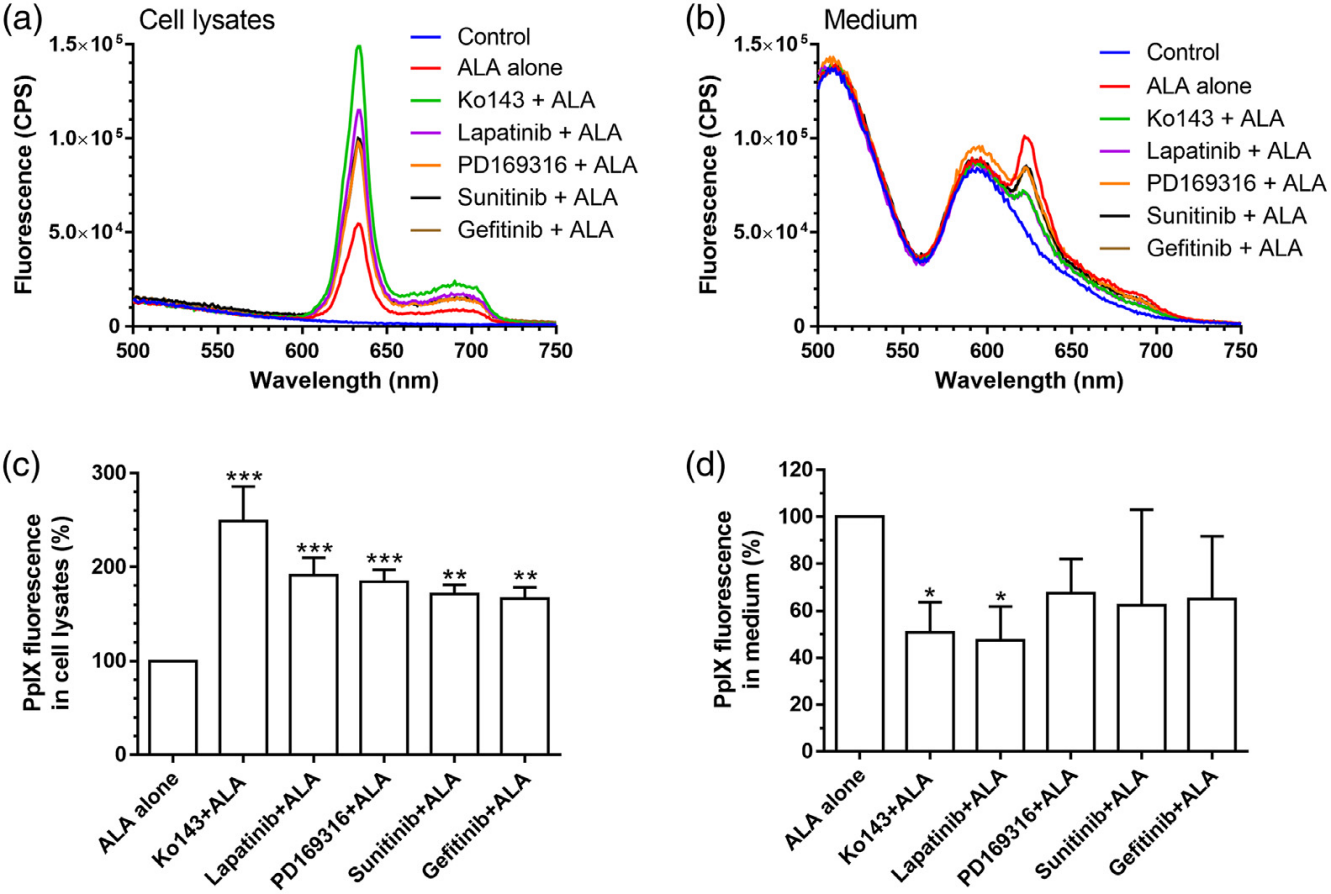
Effects of kinase inhibitors on ALA-PpIX fluorescence emission in cell lysates and culture medium. MDA-MB-231 cells were incubated with 1 mM ALA alone or in combination with Ko143 or kinase inhibitors (all at 1μM) for 4 h. Control received no treatment. PpIX fluorescence spectra of (a) cell lysates and (b) medium were measured with a spectrofluorometer. PpIX fluorescence in (c) cell lysates and (d) medium was quantified and normalized to the fluorescence of ALA treatment alone to show the percent change. Data are presented as mean ± standard deviation (SD) from at least three independent experiments. *P<0.05, **P<0.01, ***P<0.001, compared with ALA alone.

Spectrofluorometric analysis showed that incubation with ALA for 4 h resulted in PpIX accumulation in MDA-MB-231 tumor cell lysates [Fig. 1(a)]. PpIX fluorescence was also detected in cell culture medium [Fig. 1(b)], suggesting PpIX efflux transport by tumor cells. Treatment with all four kinase inhibitors as well as ABCG2 inhibitor Ko143 significantly increased PpIX fluorescence in the cell lysates [Fig. 1(c)]. Lapatinib and Ko143 significantly reduced PpIX efflux into the medium [Fig. 1(d)]. Treatment with three other kinase inhibitors (PD169316, sunitinib, and gefitinib) also showed the trend of reducing PpIX fluorescence in the medium, although the results were not statistically significant as indicated by one-way ANOVA test.

**Fig. 2:**
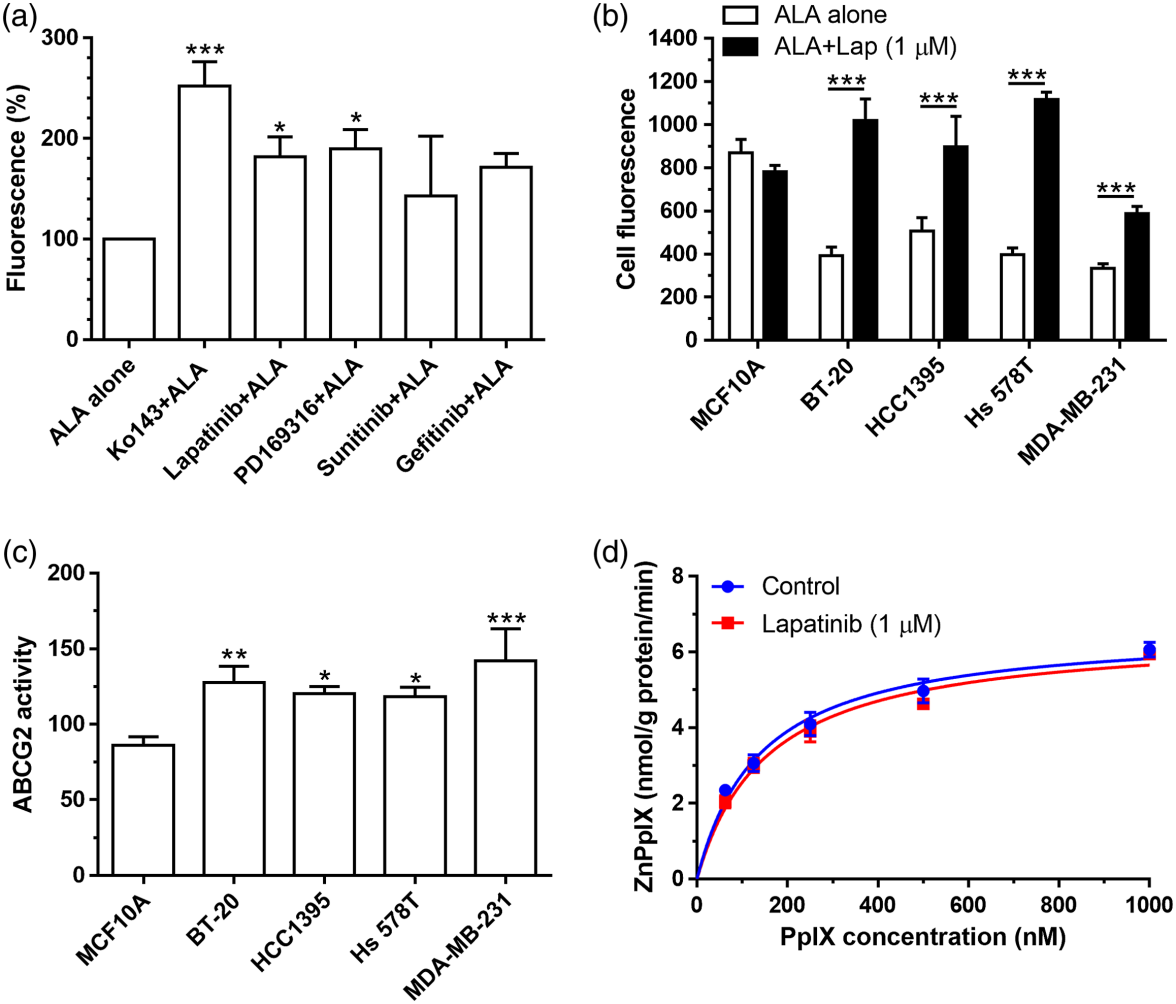
(a) Effects of kinase inhibitors on ALA-PpIX fluorescence by flow cytometry. MDA-MB-231 cells were incubated with 1 mM ALA alone or in combination with Ko143 or kinase inhibitors (all at 1  μM) for 4 h. PpIX fluorescence was measured with a flow cytometer and normalized to the fluorescence of ALA alone to show the percent change. *P<0.05, ***P<0.001, compared with ALA alone. (b) Effects of lapatinib on ALA-PpIX fluorescence. Four TNBC cell lines and nontumor MCF10A cells were incubated with 1 mM ALA alone or in combination with 1  μM lapatinib for 4 h. PpIX fluorescence was measured with a flow cytometer. ***P<0.001, compared with the corresponding ALA treatment alone. (c) ABCG2 transporter activity of MCF10A and TNBC cell lines. *P<0.05, **P<0.01, ***P<0.001, compared with MCF10A. (d) Effects of lapatinib (1  μM for 4 h) on FECH activity in MDA-MB-231 cells. Control received no treatment. All data are presented as mean ± SD from at least three independent experiments.

Effects of kinase inhibitors on ALA-PpIX fluorescence were also examined by flow cytometry. Treatment of MDA-MB-231 cells with Ko143, lapatinib, and PD169316 led to significant increase in the FL3 channel fluorescence [Fig. 2(a)]. Fluorescence was also increased after treatment with sunitinib and gefitinib, although they were not statistically significant. Because lapatinib significantly increased ALA-PpIX fluorescence in cell lysates and reduced PpIX efflux in the medium, it was chosen for the evaluation of PpIX fluorescence enhancement in a panel of TNBC cell lines and a nontumor breast epithelial cell line MCF10A. Figure 2(b) shows that lapatinib significantly enhanced ALA-PpIX fluorescence in all four TNBC cell lines, but not in MCF10A cells. All four TNBC cell lines were found to exhibit higher ABCG2 activity than MCF10A cells [Fig. 2(c)]. FECH enzymatic activity assay showed that lapatinib treatment had no significant effect on FECH activity [Fig. 2(d)].

### 3.2 Kinase Inhibitors Increased ALA-PpIX Fluorescence in Mitochondria

**Fig. 3:**
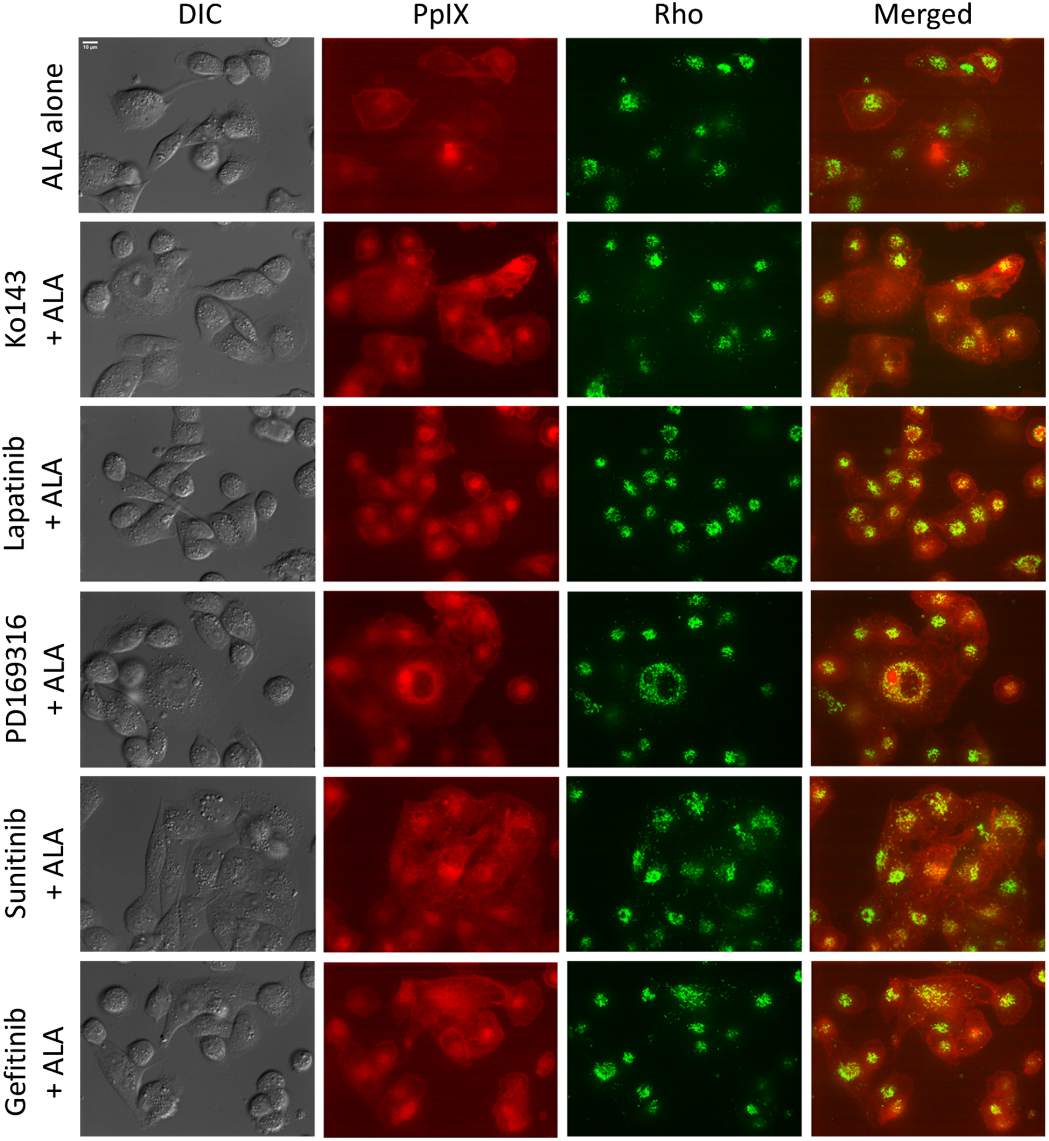
Effects of kinase inhibitors on PpIX intracellular localization. MDA-MB-231 cells were incubated with 1 mM ALA alone or in combination with Ko143 or kinase inhibitors (all at 1  μM) for 4 h and imaged with a confocal fluorescence microscope. Mitochondria were marked by rhodamine 123 (250 ng/ml) added into cell culture medium at 30 min before imaging. Bar, 10  μm.

**Fig. 4:**
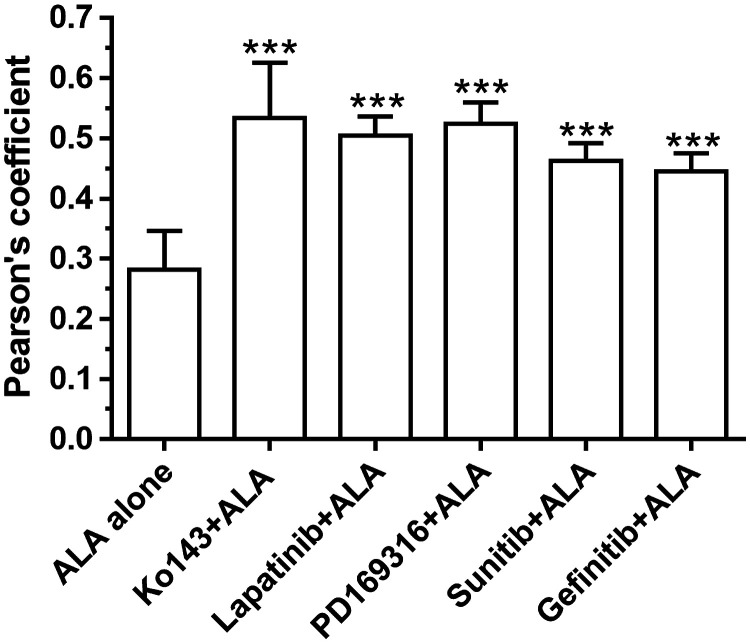
The colocalization between PpIX and Rho fluorescence was determined by Pearson’s coefficient analyzed with the NIH Image J software. Data are presented as mean ± SD from six images. ***P<0.001, compared with ALA treatment alone.

ALA-PpIX fluorescence was imaged with a confocal fluorescence microscope after 4 h incubation with ALA alone or in combination with Ko143 or kinase inhibitors. PpIX fluorescence was weak and diffuse in MDA-MB-231 cells treated with ALA alone (Fig 3). In contrast, treatment with Ko143 as well as kinase inhibitors greatly enhanced ALA-PpIX fluorescence, particularly in the mitochondria (highlighted by Rho fluorescence). Colocalization analysis of PpIX and Rho fluorescence images indicate that Ko143 and kinase inhibitors significantly increased PpIX accumulation in the mitochondria (Fig. 4).

### 3.3 Kinase Inhibitors Sensitized TNBC Cells to ALA-PDT by Inducing Apoptosis

**Fig. 5:**
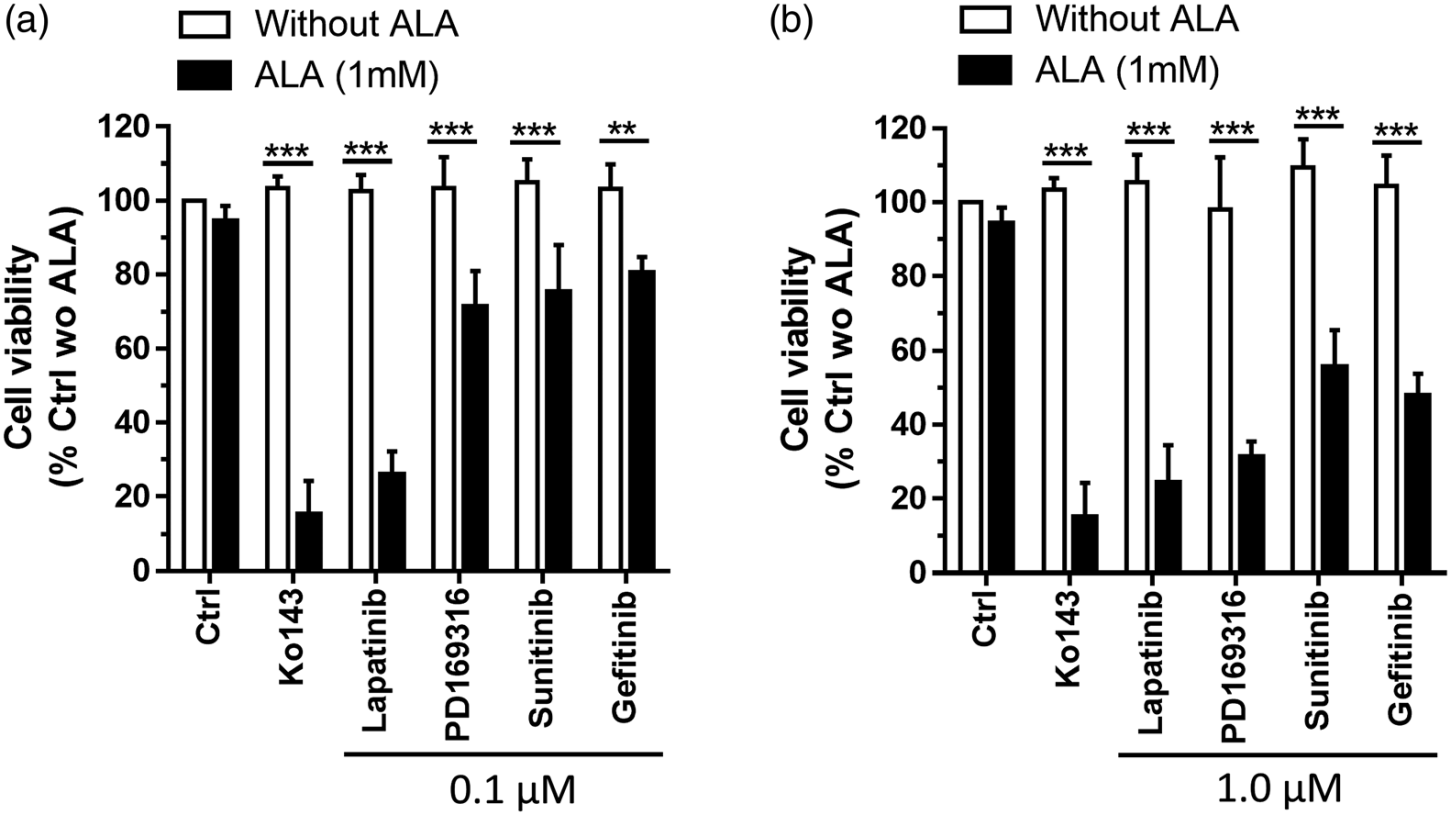
Effects of ALA-PDT alone, kinase inhibitors alone, and ALA-PDT in combination with kinase inhibitors on cell viability. MDA-MB-231 cells were incubated with ALA (1 mM) alone, kinase inhibitors alone at (a) 0.1  μM or (b) 1.0  μM, or ALA in combination with kinase inhibitors for 4 h. Cells were then treated with $3\;{J/\mathrm{cm}}^{2}$ dose of light ($5\;\mathrm{mW}/{\mathrm{cm}}^{2}$ irradiance of 633 nm light for 10 min). Cell viability was determined at 24 h after treatment. In both (a) and (b), the dose of Ko143 was 1.0  μM. Data are presented as mean ± SD from four experiments. **P<0.01, ***P<0.001.

The cell viability of MDA-MB-231 cells after ALA-PDT alone, kinase inhibitors alone, and combination treatments was determined and shown in Fig. 5. Neither ALA-PDT alone nor kinase inhibitors alone had any significant effect on cell viability. PDT in combination with kinase inhibitors (both 0.1 and 1.0  μM) led to significant reduction in cell viability. Lapatinib was more effective in enhancing PDT than other kinase inhibitors at the concentration of 0.1  μM.

**Fig. 6:**
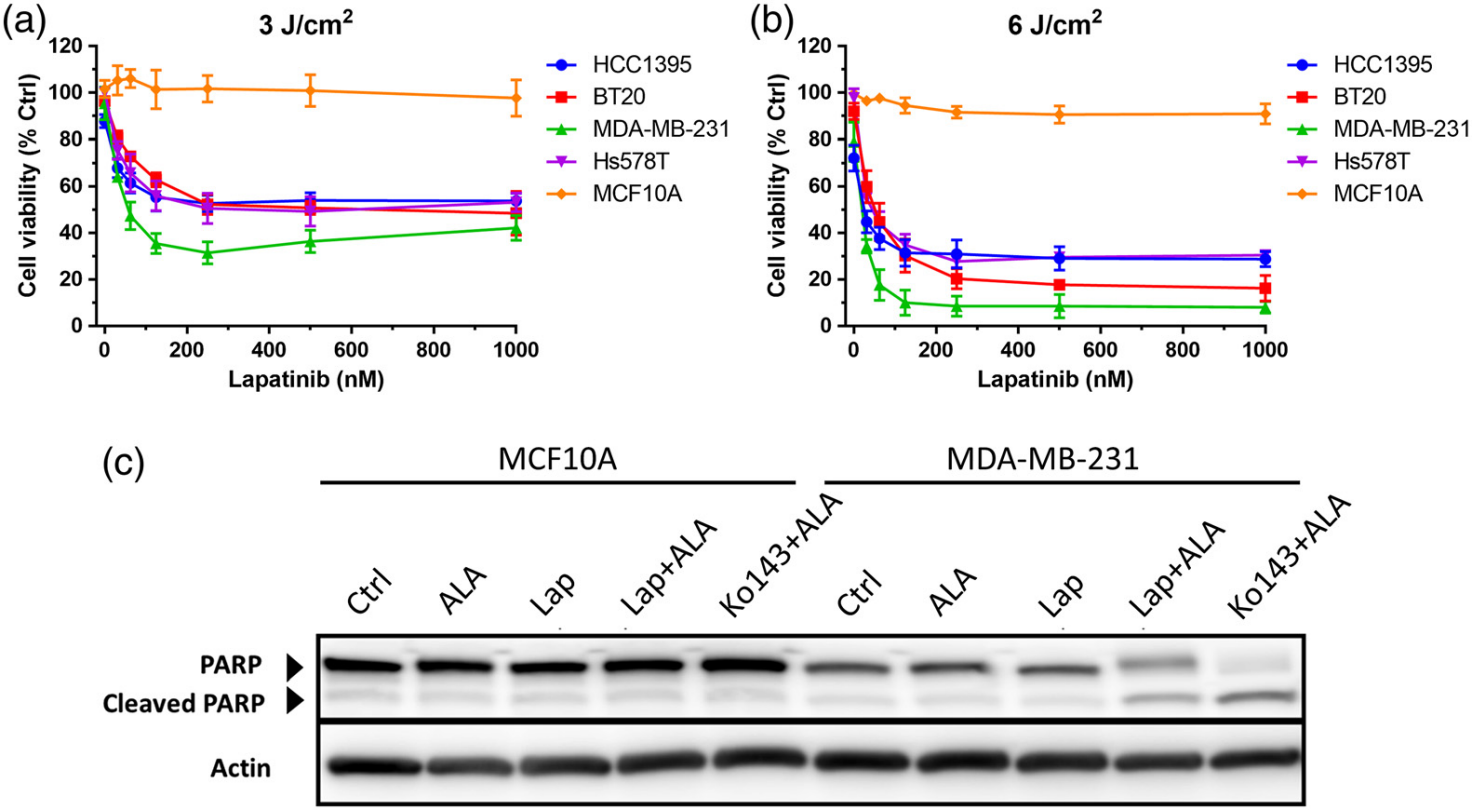
Effects of ALA-PDT in combination with lapatinib on (a), (b) cell viability and (c) apoptosis. Cells were incubated with ALA (1 mM) alone or in combination with various doses of lapatinib for 4 h. Cells were treated with either (a) $3\;{J/\mathrm{cm}}^{2}$ or (b) $6\;J/{\mathrm{cm}}^{2}$ dose of light ($5\text{-}\mathrm{mW}/{\mathrm{cm}}^{2}$ irradiance of 633 nm light). Cell viability was determined at 24 h after treatment. (c) Cell lysates were probed for PARP cleavage, the marker of apoptosis, by Western blot. MCF10A and MDA-MB-231 cells were incubated with ALA alone (1 mM), ALA in combination with lapatinib (1  μM) or with Ko143 (1  μM) for 4 h and then exposed to $3.0\;J/{\mathrm{cm}}^{2}$ light treatment. Cell lysates were prepared at 24 h after treatment for Western blot.

Effects of lapatinib in combination with ALA-PDT on cell viability were further evaluated in four TNBC cell lines and MCF10A cells. Compared with PDT alone (the first data points in figures), combination treatments resulted in significantly lower cell viability in all four TNBC cell lines but not in MCF10A cells [Fig. 6(a) and (b)]. Lapatinib caused a dose-dependent decrease in cell viability when used in combination with ALA-PDT. PDT combined with lapatinib or Ko143 induced PARP cleavage, the hallmark of apoptosis, in MDA-MB-231 cells, whereas PDT or lapatinib treatment alone did not show clear cleavage [Fig. 6(c)]. No PARP cleavage was detected after either single treatment or combination treatments in MCF10A cells.

## 4 Discussion

Effects of small molecule kinase inhibitors on ALA-PpIX fluorescence and PDT response were evaluated in TNBC cell lines in this study. The results confirmed our previous findings in renal cell carcinoma cell lines^9^ and demonstrate in TNBC cell lines that clinically approved kinase inhibitors, such as lapatinib, sunitinib, and gefitinib, increased ALA-PpIX fluorescence. As in the previous study, we found here that lapatinib was the only kinase inhibitor that significantly increased intracellular PpIX and reduced PpIX efflux. Both our previous and present studies suggest the use of lapatinib and other clinically approved kinase inhibitors for the enhancement of ALA-PpIX fluorescence.

Enhancement of PpIX by kinase inhibitors greatly sensitized TNBC cell lines to ALA-PDT, which were not sensitive to PDT alone likely due to ABCG2-mediated PpIX efflux. All four TNBC cell lines used in the study showed elevated ABCG2 activity [Fig. 2(c)]. Confocal fluorescence imaging revealed weak PpIX fluorescence, which was enhanced by ABCG2 inhibitor Ko143 and kinase inhibitors (Fig. 3). Importantly, we found that Ko143 and kinase inhibitors increased PpIX localization in mitochondria (Fig. 3 and 4). This finding is in agreement with a previous study showing that ABCG2 transporter is localized in both cell membrane and mitochondria.^19^ Since PpIX is biosynthesized inside the mitochondria, suppression of ABCG2 efflux function in mitochondria by Ko143 and kinase inhibitors resulted in PpIX accumulation in mitochondria. As mitochondria are important for initiating cell apoptosis, increased PpIX accumulation in mitochondria led to increased apoptosis induced by PDT in combination with Ko143 or lapatinib (Fig. 6).

As a dual kinase inhibitor of human epidermal growth receptors EGFR and HER2 for breast cancer treatment, lapatinib is a substrate for ABCG2 and P-glycoprotein (P-gp), another ATP-binding cassette family protein commonly involved in multidrug resistance to cancer chemotherapy.^20^ In addition to lapatinib, other tyrosine kinase inhibitors, such as imatinib and gefitinib, are ABCG2 substrates as well.^13^ Because diverse ABCG2 substrates share a common binding site in the ligand binding domain of ABCG2, they also function as competitive ABCG2 inhibitors.^21^ Such interactions between tyrosine kinase inhibitors and ATP-binding cassette efflux transporters have stimulated the use of kinase inhibitors to overcome transporter-mediated multidrug resistance to chemotherapeutic agents.^22^

PpIX is a substrate for ABCG2, but not for P-gp (ABCB1) or multidrug resistance-associated protein 1 (ABCC1).^5^ Repositioning of ABCG2-interacting kinase inhibitors for the enhancement of ALA-PpIX is important and necessary, considering that no ABCG2 inhibitor is currently available for clinical application. Tyrosine kinase inhibitors imatinib^14^ and gefitinib^15^ have been shown to enhance ALA-PpIX fluorescence and PDT due to the inhibition of ABCG2. Our previous evaluation of over 12 kinase inhibitors led to the identification of lapatinib as the most potent drug for enhancing ALA-PpIX fluorescence.^9^ This study further demonstrates the effectiveness of lapatinib in enhancing ALA-PDT by inducing apoptosis. Strong enhancement of ALA-PpIX fluorescence and PDT by lapatinib was likely due to its potent inhibition of ABCG2 activity. Lapatinib inhibited pheophorbide a-mediated transport in an ABCG2-expressing cell line with an ${\mathrm{IC}}_{50}$ of 0.08  μM, the lowest among all 11 ABCG2-interacting kinase inhibitors examined.^21^ Its ABCG2 inhibitory activity was much stronger than imatinib (${\mathrm{IC}}_{50}:0.99\;\mu M$), gefitinib (${\mathrm{IC}}_{50}:3.01\;\mu M$), and sunitinib (${\mathrm{IC}}_{50}:0.57\;\mu M$), supporting the use of lapatinib as a potent ABCG2 inhibitor to enhance ALA-PpIX fluorescence and PDT.

Interestingly, some kinase inhibitors including gefitinib and vemurafenib were shown to display off-target binding and inhibition to FECH, the enzyme catalyzing the conversion of PpIX to heme.^23^ Because the inhibition of FECH activity is known to increase PpIX fluorescence,^24^ we examined FECH activity after lapatinib treatment. Our data indicate that lapatinib at 1  μM, the highest concentration we used for the enhancement of ALA-PpIX and PDT, had no significant effect on FECH activity, which excludes the contribution of FECH inhibition to lapatinib-induced PpIX enhancement. In addition to FECH inhibition, increased activity of heme biosynthesis enzymes upstream of FECH may also lead to PpIX enhancement.^2^ Although the effect of lapatinib on those heme biosynthesis enzymes is not known, the finding that lapatinib-induced PpIX enhancement was observed only in cells lines with elevated ABCG2 activity suggests that it does not have a significant effect on those enzymes.

## 5 Summary

We have shown in this study the effectiveness of four ABCG2-interacting kinase inhibitors (lapatinib, PD169316, sunitinib, and gefitinib) in enhancing ALA-PpIX fluorescence and PDT in TNBC cell lines. These results together with our previous study demonstrate the feasibility of repurposing clinical kinase inhibitors for the therapeutic enhancement of ALA in tumors with elevated ABCG2 activity. Particularly our study revealed lapatinib as a potent agent for enhancing ALA. Lapatinib in combination with ALA significantly increased PpIX fluorescence and PDT-induced apoptosis in tumor cells that were resistant to ALA-PDT alone. Our future work will focus on determining whether this therapeutic enhancement approach leads to enhanced ALA-PpIX fluorescence and PDT response in tumor models with elevated ABCG2 activity.

## References

[1] Hadjipanayis C. G. Stummer W., “5-ALA and FDA approval for glioma surgery,” J. Neurooncol. 141, 479–486 (2019). 10.1007/s11060-019-03098-y 30644008 PMC6445645

[2] Yang X. et al., “Aminolevulinic acid-based tumor detection and therapy: molecular mechanisms and strategies for enhancement,” Int. J. Mol. Sci. 16, 25865–25880 (2015).1422-0067 10.3390/ijms161025865 26516850 PMC4632830

[3] Tarstedt M. et al., “Aminolevulinic acid and methyl aminolevulinate equally effective in topical photodynamic therapy for non-melanoma skin cancers,” J. Eur. Acad. Dermatol. Venereol. 30, 420–423 (2016). JEAVEQ 0926-9959 10.1111/jdv.13558 26841041

[4] Stummer W. et al., “Fluorescence-guided surgery with 5-aminolevulinic acid for resection of malignant glioma: a randomised controlled multicentre phase III trial,” Lancet Oncol. 7, 392–401 (2006). LOANBN 1470-2045 10.1016/S1470-2045(06)70665-9 16648043

[5] Robey R. W. et al., “ABCG2-mediated transport of photosensitizers: potential impact on photodynamic therapy,” Cancer Biol. Ther. 4, 195–202 (2005). 10.4161/cbt.4.2.1440 15684613

[r6] Barron G. A. Moseley H. Woods J. A., “Differential sensitivity in cell lines to photodynamic therapy in combination with ABCG2 inhibition,” J. Photochem. Photobiol. B 126, 87–96 (2013). JPPBEG 1011-1344 10.1016/j.jphotobiol.2013.07.003 23911860

[7] Muller P. et al., “ABCG2 influence on the efficiency of photodynamic therapy in glioblastoma cells,” J. Photochem. Photobiol. B 210, 111963 (2020). JPPBEG 1011-1344 10.1016/j.jphotobiol.2020.111963 32795847

[8] Palasuberniam P. et al., “ABCG2 transporter inhibitor restores the sensitivity of triple negative breast cancer cells to aminolevulinic acid-mediated photodynamic therapy,” Sci. Rep. 5, 13298 (2015). SRCEC3 2045-2322 10.1038/srep13298 26282350 PMC4539603

[9] Howley R. et al., “Evaluation of aminolevulinic acid-mediated protoporphyrin IX fluorescence and enhancement by ABCG2 inhibitors in renal cell carcinoma cells,” J. Photochem. Photobiol. B 211, 112017 (2020). JPPBEG 1011-1344 10.1016/j.jphotobiol.2020.112017 32919173 PMC7545695

[10] Allen J. D. et al., “Potent and specific inhibition of the breast cancer resistance protein multidrug transporter *in vitro* and in mouse intestine by a novel analogue of fumitremorgin C,” Mol. Cancer Ther. 1, 417–425 (2002). 12477054

[11] Weidner L. D. et al., “The inhibitor Ko143 is not specific for ABCG2,” J. Pharmacol. Exp. Ther. 354, 384–393 (2015). JPETAB 0022-3565 10.1124/jpet.115.225482 26148857 PMC4538874

[12] Robey R. W. et al., “Revisiting the role of ABC transporters in multidrug-resistant cancer,” Nat. Rev. Cancer 18, 452–464 (2018). NRCAC4 1474-175X 10.1038/s41568-018-0005-8 29643473 PMC6622180

[13] Mao Q. Unadkat J. D., “Role of the breast cancer resistance protein (BCRP/ABCG2) in drug transport—an update,” AAPS J. 17, 65–82 (2015). 10.1208/s12248-014-9668-6 25236865 PMC4287283

[14] Liu W. et al., “The tyrosine kinase inhibitor imatinib mesylate enhances the efficacy of photodynamic therapy by inhibiting ABCG2,” Clin. Cancer Res. 13, 2463–2470 (2007). 10.1158/1078-0432.CCR-06-1599 17438106

[15] Sun W. et al., “Gefitinib enhances the efficacy of photodynamic therapy using 5-aminolevulinic acid in malignant brain tumor cells,” Photodiagn. Photodyn. Ther. 10, 42–50 (2013). 10.1016/j.pdpdt.2012.06.003 23465372

[16] Kumari M. Krishnamurthy P. T. Sola P., “Targeted drug therapy to overcome chemoresistance in triple-negative breast cancer,” Curr. Cancer Drug Targets 20, 559–572 (2020). 10.2174/1568009620666200506110850 32370716

[17] Yang X. et al., “Her2 oncogene transformation enhances 5-aminolevulinic acid-mediated protoporphyrin IX production and photodynamic therapy response,” Oncotarget 7, 57798–57810 (2016). 10.18632/oncotarget.11058 27527860 PMC5295390

[18] Fateye B. et al., “Combination of phosphatidylinositol 3-kinases pathway inhibitor and photodynamic therapy in endothelial and tumor cells,” Photochem. Photobiol. 88, 1265–1272 (2012). PHCBAP 0031-8655 10.1111/j.1751-1097.2012.01160.x 22506666

[19] Kobuchi H. et al., “Mitochondrial localization of ABC transporter ABCG2 and its function in 5-aminolevulinic acid-mediated protoporphyrin IX accumulation,” PLoS One 7, e50082 (2012). POLNCL 1932-6203 10.1371/journal.pone.0050082 23189181 PMC3506543

[20] Polli J. W. et al., “The role of efflux and uptake transporters in [N-{3-chloro-4-[(3-fluorobenzyl)oxy]phenyl}-6-[5-({[2-(methylsulfonyl)ethyl]amino}methyl)-2-furyl]-4-quinazolinamine (GW572016, lapatinib) disposition and drug interactions,” Drug Metab. Dispos. 36, 695–701 (2008). DMDSAI 0090-9556 10.1124/dmd.107.018374 18216274

[21] Gose T. et al., “ABCG2 requires a single aromatic amino acid to ‘clamp’ substrates and inhibitors into the binding pocket,” FASEB J. 34, 4890–4903 (2020). FAJOEC 0892-6638 10.1096/fj.201902338RR 32067270 PMC8317467

[22] Beretta G. L. et al., “Overcoming ABC transporter-mediated multidrug resistance: the dual role of tyrosine kinase inhibitors as multitargeting agents,” Eur. J. Med. Chem. 142, 271–289 (2017). EJMCA5 0223-5234 10.1016/j.ejmech.2017.07.062 28851502

[23] Klaeger S. et al., “Chemical proteomics reveals ferrochelatase as a common off-target of kinase inhibitors,” ACS Chem. Biol. 11, 1245–1254 (2016). 10.1021/acschembio.5b01063 26863403

[24] Kemmner W. et al., “Silencing of human ferrochelatase causes abundant protoporphyrin-IX accumulation in colon cancer,” FASEB J. 22, 500–509 (2008). FAJOEC 0892-6638 10.1096/fj.07-8888com 17875605

